# Author response to: Letter to the Editor on ‘Adherence to oral zinc supplementation in the management of acute diarrhoeal disease among under-5 children: A systematic review and meta-analysis’

**DOI:** 10.1017/S0950268826101459

**Published:** 2026-07-08

**Authors:** Somen Kumar Pradhan, Ashutosh Panda, Sanghamitra Pati, Jaya Singh Kshatri

**Affiliations:** 1 Community Medicine, Maharaja Krishna Chandra Gajapati Medical College and Hospital, India; 2 Indian Council of Medical Research, Department of Health Research, New Delhi, India; 3 ICMR-RMRC Bhubaneswar, India; 4 Maastricht University, Netherlands

Dear Editor,

We thank *Katkuri et al.* for their careful reading of our article and for their constructive engagement with our work [1]. We appreciate their recognition of the importance of adherence research in childhood diarrhoea and of the methodological strengths of our review. We address their specific methodological concerns below.

## Inclusion of interventional design

The authors presented their concern regarding the inclusion of a study by *Nuzhat et al.*, described by its authors as a ‘prospective open-label interventional study’ [2]. However, a detailed methodological appraisal indicates that, despite this terminology, the study does not meet the defining criteria of an interventional trial. The study did not involve randomization, allocation to alternative interventions, or the testing of any adherence-enhancing strategies; rather, all participants received zinc as part of routine diarrhoeal case management, with the investigators solely documenting completion of therapy. The outcome of interest was descriptive and behavioural (treatment adherence) rather than comparative efficacy, and no protocol-driven manipulation of exposure was undertaken. In epidemiological terms, this design corresponds to a prospective observational cohort study conducted in a real-world setting instead of an experimental trial [3]. As our meta-analysis synthesized prevalence of adherence, not treatment effects, inclusion of such real-world observational data is consistent with established guidance for prevalence meta-analysis. We analysed proportions rather than effect sizes and conducted sensitivity analyses, which showed that excluding this study did not materially change the pooled estimates. While we did not modify or reinterpret the study design as reported by the original authors, our synthesis was based solely on the observational adherence data presented in the study [4]. Its inclusion was therefore methodologically appropriate for estimating adherence prevalence and does not compromise the validity or interpretability of our findings.

## Use and scoring of the NHLBI Risk-of-Bias Tool

The authors have also commented regarding our application of the National Heart, Lung, and Blood Institute (NHLBI) Quality Assessment Tool. We acknowledge that the tool’s developers caution against creating psychometric summary scores and against assuming equal weighting across domains. Accordingly, our intention was not to use numerical scoring to weight studies or influence pooled estimates, but to provide a transparent overview of methodological limitations [5]. We have explicitly reported the selected domains (D1–D6) and their coding in the Methods section, with each item scored dichotomously (presence of bias = 1; absence of bias = 0). The overall categorization (low risk, some concern, high risk) was based on the cumulative number of domains indicating potential bias, such that higher scores reflected a greater number of identified methodological limitations rather than a psychometric weighting of study quality. Importantly, no study was excluded or down-weighted based on this classification. The guidance site on the NHLBI Quality Assessment Tool for Observational Cohort and Cross-Sectional Studies explicitly states that items not applicable to certain designs (e.g., cross-sectional studies) may be marked as ‘Not Applicable’, and that ‘researchers may determine their own parameters for making judgments’ [6]. Thus, while the tool does not prescribe a mandatory scoring framework, it permits flexible, study-specific application. Our approach, therefore, aligns with the NHLBI guidance and was used solely to contextualize internal validity. Furthermore, as explained earlier, the study described as ‘interventional’ lacks randomization or experimental manipulation and is analytically observational, supporting the appropriateness of domain-level assessment.

## High heterogeneity and interpretation of pooled estimates

The authors further highlighted the extremely high heterogeneity (*I*^2^ ≈ 99%), and we explicitly cautioned against over-interpretation of the pooled estimates. The aim of our meta-analysis was not to derive a single ‘definitive’ prevalence of adherence, but to quantify variability and highlight operational challenges in zinc programme implementation across heterogeneous settings. To address this, we conducted separate analyses for 10-day and 14-day regimens, performed leave-one-out sensitivity analyses, and interpreted findings in conjunction with contextual determinants reported in the primary studies [7]. Our results, therefore, emphasize consistent patterns of suboptimal adherence, particularly for longer regimens rather than reliance on a single summary estimate. This approach is consistent with current methodological guidance that meta-analysis in the presence of substantial heterogeneity can remain informative for hypothesis generation and policy signalling rather than precise estimation [8].

## Variable definitions of adherence

Finally, the authors also mentioned that adherence was treated as a homogeneous construct, despite variability in its definition across studies, including completion of 10-day or 14-day regimens and caregiver-reported duration of use. To address this conceptual heterogeneity, we explicitly stratified analyses by treatment duration, thereby avoiding inappropriate pooling across fundamentally different adherence definitions. Moreover, our narrative synthesis mapped determinants such as early discontinuation due to symptom resolution, vomiting, caregiver perceptions, and provider counselling, factors that reflect distinct behavioural mechanisms underlying non-adherence. To enhance transparency, we have also presented the definitions of diarrhoea and zinc adherence used in the included studies in Appendix D of the original article. Rather than obscuring these differences, our approach highlights the multidimensional nature of adherence in community diarrhoea management [9].

## Conclusion

We appreciate the thoughtful critique offered by *Katkuri et al.* and agree that adherence research in childhood diarrhoea demands careful methodological consideration. Our study provides the first structured synthesis of adherence patterns, demonstrating consistently suboptimal completion, particularly for 14-day regimens, across diverse LMIC settings. Importantly, by analysing 10-day and 14-day courses separately, our work also seeks to clarify the programmatic ambiguity arising from the WHO recommendation of ‘10–14 days’ of zinc therapy, highlighting how regimen duration itself influences adherence. We believe that, when interpreted appropriately, our findings contribute important evidence for programme design, formulation strategies, and caregiver-provider communication, and we welcome continued scholarly dialogue to strengthen the evidence base for zinc implementation in child health.

## Data Availability

No new data were generated or analysed for this author response. The content is based solely on previously published studies and the methodological interpretation of the authors’ original work.

## References

[1] Pradhan SK, et al. (2025) Adherence to oral zinc supplementation in the management of acute diarrhoeal disease among under-5 children: A systematic review and meta-analysis. Epidemiology and Infection 153. 10.1017/S0950268825100733. PMC12641308 41178278

[2] Nuzhat S, et al. (2022) New formulation zinc sulphate acceptability and adherence in children with acute diarrhoea: A prospective, open-label, interventional study in Bangladesh. Journal of Paediatrics and Child Health 58(7), 1215–1220. 10.1111/jpc.15953. 35348269

[3] Soumerai SB, Starr D and Majumdar SR (2015) How do you know which health care effectiveness research you can trust? A guide to study Design for the Perplexed. Preventing Chronic Disease 12, E101. 10.5888/pcd12.150187. 26111157 PMC4492215

[4] Shrier I, et al. (2007) Should meta-analyses of interventions include observational studies in addition to randomized controlled trials? A critical examination of underlying principles. American Journal of Epidemiology 166(10), 1203–1209. 10.1093/AJE/KWM189. 17712019

[5] Mamikutty R, Aly AS and Marhazlinda J (2021) Selecting risk of bias tools for observational studies for a systematic review of anthropometric measurements and dental caries among children. International Journal of Environmental Research and Public Health 18(16), 8623. 10.3390/ijerph18168623. 34444374 PMC8391268

[6] National Heart, Lung, and Blood Institute, (NHLBI) 2021 *Quality Assessment Tool for Observational Cohort and Cross-Sectional Studies.* https://www.nhlbi.nih.gov/health-topics/study-quality-assessment-tools.

[7] Schroll JB, Moustgaard R and Gøtzsche PC (2011) Dealing with substantial heterogeneity in Cochrane reviews. Cross-sectional study. BMC Medical Research Methodology 11(1), 22. 10.1186/1471-2288-11-22. 21349195 PMC3056846

[8] Kang H and Choi GJ (2025) Heterogeneity in meta-analyses: An unavoidable challenge worth exploring. Korean Journal of Anesthesiology 78(4), 301–314. 10.4097/kja.25001. 39956636 PMC12326561

[9] Bentley S, et al. (2024) Improving assessment of adherence behaviors and drivers: Targeted literature review and concept elicitation interviews in multiple countries and disease populations. Patient Preference and Adherence, 1231–1242. 10.2147/PPA.S433662. 38911591 PMC11192640

